# Vascular Polyurethane Prostheses Modified with a Bioactive Coating—Physicochemical, Mechanical and Biological Properties

**DOI:** 10.3390/ijms222212183

**Published:** 2021-11-10

**Authors:** Aleksandra Kuźmińska, Aleksandra Wojciechowska, Beata A. Butruk-Raszeja

**Affiliations:** Biomedical Engineering Laboratory, Faculty of Chemical and Process Engineering, Warsaw University of Technology, Warynskiego 1, 00-645 Warsaw, Poland; aleksandra.wojciechowska2.stud@pw.edu.pl (A.W.); Beata.Raszeja@pw.edu.pl (B.A.B.-R.)

**Keywords:** phase-inversion technique, vascular prosthesis, polyurethane, surface modification, hemocompatibility, endothelial cell, REDV, YIGSR

## Abstract

This study describes a method for the modification of polyurethane small-diameter (5 mm) vascular prostheses obtained with the phase inversion method. The modification process involves two steps: the introduction of a linker (acrylic acid) and a peptide (REDV and YIGSR). FTIR and XPS analysis confirmed the process of chemical modification. The obtained prostheses had a porosity of approx. 60%, Young’s Modulus in the range of 9–11 MPa, and a water contact angle around 40°. Endothelial (EC) and smooth muscle (SMC) cell co-culture showed that the surfaces modified with peptides increase the adhesion of ECs. At the same time, SMCs adhesion was low both on unmodified and peptide-modified surfaces. Analysis of blood-materials interaction showed high hemocompatibility of obtained materials. The whole blood clotting time assay showed differences in the amount of free hemoglobin present in blood contacted with different materials. It can be concluded that the peptide coating increased the hemocompatibility of the surface by increasing ECs adhesion and, at the same time, decreasing platelet adhesion. When comparing both types of peptide coatings, more promising results were obtained for the surfaces coated with the YISGR than REDV-coated prostheses.

## 1. Introduction

Cardiovascular diseases are still the leading cause of death in the world [1]. Therefore, research is continuously carried out to produce vascular prostheses with small diameters (<6 mm), especially since autologous transplants are often not possible [2]. However, it is rare for a material to exhibit appropriate physicochemical and mechanical properties and the expected biocompatibility in practice [3]. Therefore, polymers with suitable mechanical and physical parameters are subject to surface modification, which does not change the base material’s mechanical properties [4]. The purpose of the modification is to obtain a biocompatible structure or give the surface the desired physiological activity. The type of material surface modification used is closely related to its intended use and period of use [5]. Bioactive coatings are characterized by the lack of toxicity and no negative reaction of the body in long-term contact with living tissue and body fluids [6]. These materials, due to biochemical reactions, interact with the surrounding environment [7]. They can activate a cascade of biological events that ultimately contribute to regeneration or allow tissue replacement while maintaining their full functionality [8]. The modified material’s necessary properties are biocompatibility, no allergic or toxic reactions, adequate mechanical strength, the ability for cells adhesion, and a lack or reduction of blood coagulation [9,10]. The prevention of blood clots and the maintenance of the body’s hemostasis becomes crucial [11]. This is especially important in small diameter vascular prostheses, where the clot can lead to the closure of the scaffold’s lumen [12]. The most commonly used polymers for vascular prostheses production are polyurethanes [13,14], known to have a high level of hemocompatibility [15]. Yet, it may be destructive to platelets. Therefore, surface modification represents an important issue.

One of the methods of surface modification is the chemical introduction of functional groups. Such groups (e.g., carboxyl) can be further reacted, thanks to which bioactive particles can be introduced onto the surfaces [16]. For example, acrylic acid can be used as a linker [17,18]. Biomimetic materials are able to cause specific cellular responses [16,19]. For endothelialization, ECM-derived short peptide sequences that selectively promote the adhesion and proliferation of endothelial cells are most promising [20,21]. Tetrapeptide REDV is a fibronectin-derived peptide, known for its adhesive properties. It allows endothelial cells (ECs) attachment and proliferation, but limits the adhesion of vascular smooth muscle [22,23]. YIGSR is not as selective as REDV; it also promotes adhesion of both ECs and smooth muscle cells [24]. Both peptide sequences inhibit platelet adhesion [25,26].

Phase inversion is not the most common technique in the literature for the production of vascular prostheses. However, with this method, after selecting the optimal process parameters, it is possible to create a structure with an appropriate morphology and mechanical properties [27,28,29].

The study aimed to obtain a tissue scaffold to use as a vascular prosthesis using the phase inversion method. In previous studies, the manufacturing parameters were selected to receive the material with the desired surface morphology [30]. The next step described here was surface modification with a previously developed method [31,32], albeit with the process parameters adjusted for the material produced by the phase inversion. Such modification increases surface wettability, resulting in better cell adhesion. In the case of the modification of flat structures, an increase in bio- and hemocompatibility has been proven. Previous works have focused on modifying other forms of materials prepared by varied techniques (foil via solvent casting, fibers via solution blow spinning) from different types of polyurethane, with a different geometry (flat discs) and surface morphology. Moreover, the reaction was carried out with different process parameters (higher concentrations of reagents, different temperatures). This work focuses on the possibility of modifying the cylindrical structure produced by a method not studied before, namely the phase inversion technique. It is worth emphasizing that prostheses obtained by this method have completely different properties than the polyurethane solid discs or fibers. Moreover, the influence of the modification on both surface morphology and mechanical properties was investigated. The study aimed to examine the effect of surface modifications on the biological properties of the produced material. The selectivity of the REDV and YIGSR sequences on the cells of endothelial and muscle cells was investigated. The introduction of the peptide sequences on the interaction of the material with blood was also assessed with platelet adhesion and whole blot clotting time assays. 

## 2. Results

### 2.1. Surface Morphology

**Figure 1:**
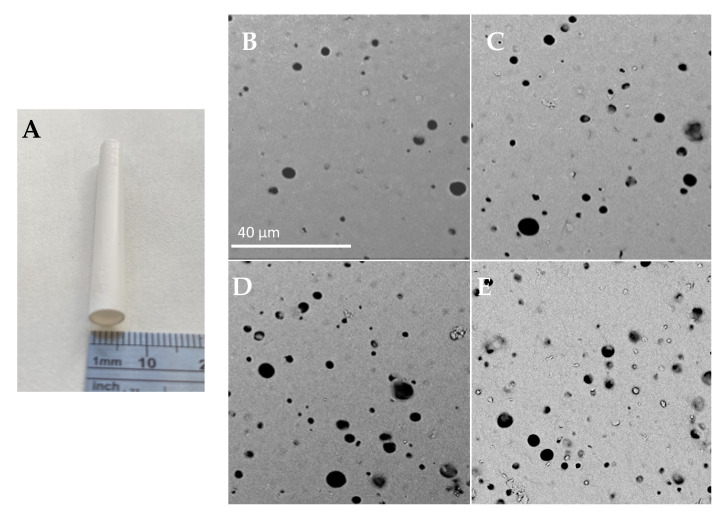
(**A**) Macroscopic images of prosthesis’; Morphology of prosthesis’ internal surface, (**B**): PU, (**C**): PU_AA, (**D**): PU_REDV, (**E**): PU_YIGSR; scale bar: 40 µm.

The studies focused on the internal surface, which will have constant contact with cells and blood, assuming the use as a vascular prosthesis. Figure 1 presents a macroscopic image of the manufactured structure and representative SEM photographs of the obtained materials’ internal surface. All materials had inner surfaces which were homogeneously porous with spherical pores. The pores were round and similar in shape, and the surface was smooth with single artifacts. The modification did not influence the prosthesis’ wall thickness; for all materials, it was approximately 230 μm, with no statistical difference between tested materials’ variants. 

**Figure 2:**
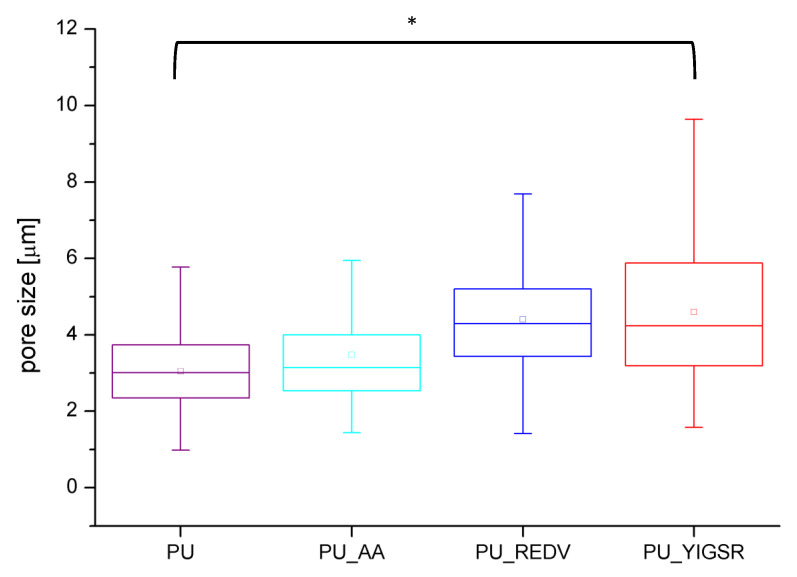
Pore size distribution for obtained materials (n = 100), *: *p* < 0.05.

The modification process extended the range of surface pore sizes (Figure 2). The average surface pores size has significantly increased (*p* < 0.05) (Table 1)—from 3.0 ± 1.0 μm for unmodified PU to 4.4 ± 1.5 and 4.6 ± 1.9 for PU_REDV and PU_YIGSR. Similarly, an increase in maximum (from 5.8 μm for PU to 9.5/10.1 μm for PU_REDV/PU_YIGSR) and minimal (from 1 μm for PU to 1.4/1.6 μm for PU_REDV/PU_YIGSR) size was observed. 

### 2.2. Porosity

In Table 1, the basic properties of obtained materials are presented, including porosity results. The modification process increased the prostheses’ porosity by approximately 13 percentage points, from 56 ± 2% for PU to 62 ± 6%/63 ± 3% for PU_REDV/PU_YIGSR. The difference was statistically significant (*p* < 0.05). This is related to an increase in the size of surface pores. No statistically significant differences in porosity between PU_AA, PU_REDV, PU_YIGSR variants were observed.

### 2.3. Mechanical Testing

Table 1 presents the mechanical properties of analyzed materials. Unmodified PU shows the lowest Young’s modulus (YM). The modification process increased YM values. Differences between modified variants were not statistically significant (*p* > 0.05). An inverse relationship was observed for tensile strength and elongation at the break–values statistically significantly decreased (*p* > 0.05). In the case of PU_AA, both of these parameters had the lowest values. Values for peptide-modified materials didn’t differ statistically significantly between themselves.

### 2.4. Chemical Characterization

**Figure 3:**
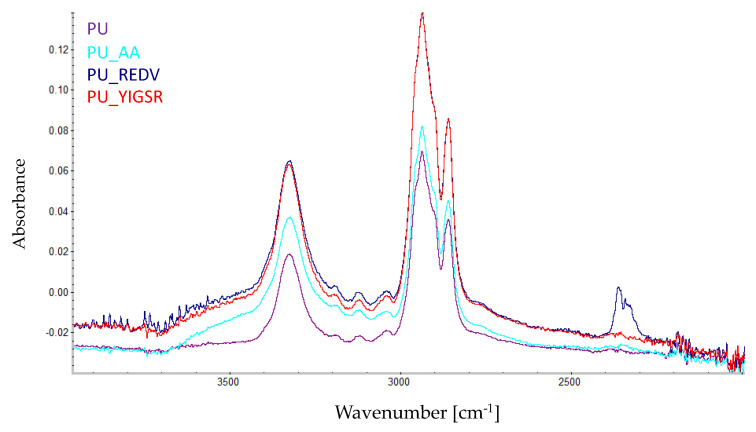
FTIR spectra recorded for the modified surfaces after each modification step.

The modification process was monitored by FTIR analysis (Figure 3). The occurrence of surface modification is confirmed by changes in the ~3600–3000 cm^−1^ region of the spectrum. In the PU_AA is a visible higher peak at ~3300 cm^−1^. For PU_REDV and PU_YIGSR peak at ~3300 cm^−1^ is higher than for PU_AA. Moreover, at 3700–3400 cm^−1^, stretching vibration bands referring to NH_2_, –OH, and –C=O groups are visible [33,34]. 

**Figure 4:**
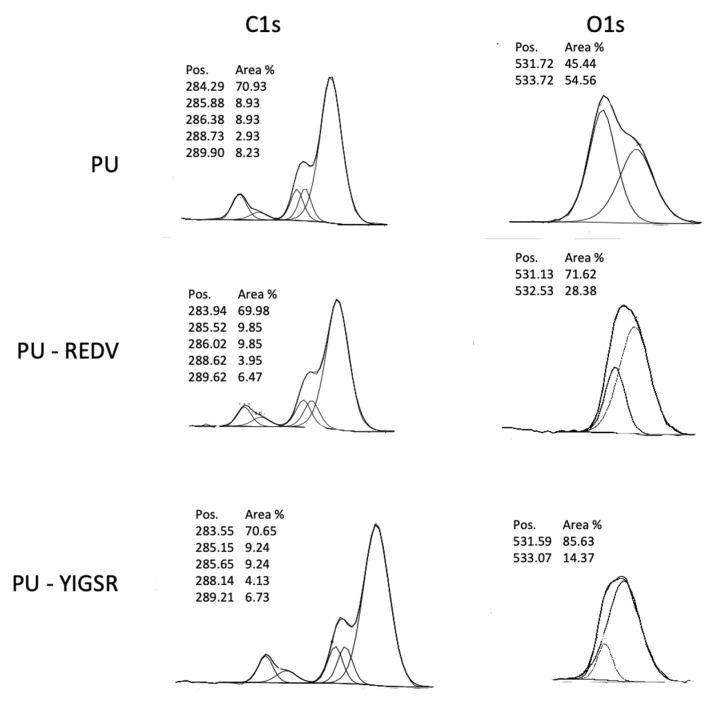
X-ray photoelectron spectroscopy (XPS) spectra of carbon C1s and O1s.

XPS analysis enabled monitoring of the subsequent steps of the modification reaction. XPS spectra of C1s and O1s are presented in Figure 4. In the case of the unmodified PU, the C1s spectrum can be curve-fitted into five peaks. The four peaks with BEs of about 284.29, 285.88, 286.38, and 288.73 eV are attributable to the C–H, C–N, C–O, and O–(C=O)–N species, which is following data presented for polyurethanes [35]. The fifth peak with a high BE of 289.9 eV corresponds to the carbonate group (O–(C=O)–O) [36] of ChronoFlex, which is a carbonate-based polyurethane. After conjugation with peptide, both in case of PU_REDV and PU_YIGSR, peak with BEs of about 288 eV slightly increases with can be associated with the introduction of amide group [37]. Greater changes are visible in the O1s spectrum O1s. The spectrum for PU was separated into two components corresponding to the binding energy ~531 eV (double-bonded oxygen) and ~533 eV (single-bonded oxygen). In the case of PU_REDV/PU_YIGSR, two peaks also appeared. However, the intensity of peaks corresponded with double-bonded oxygen strongly increased. 

### 2.5. Water Contact Angle

**Figure 5:**
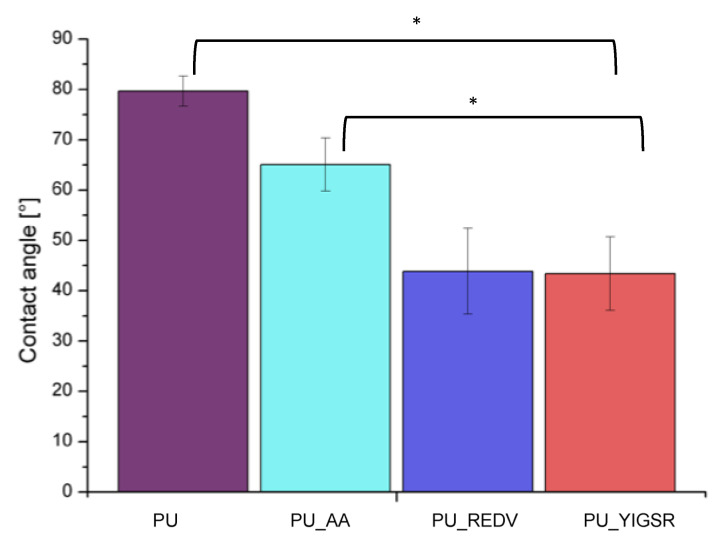
The wettability results (MV ± SD, n = 4) *: *p* < 0.05.

The results of surface wettability measurements are presented in Figure 5. The unmodified PU has WCA = 79.7 ± 3.0°, which means it is slightly hydrophilic. After modification, the wettability of the surface increased. WCA values for all modified surfaces statistically significantly differed (*p* < 0.05) from the unmodified PU WCA value. The reduction of WCA confirms that both steps of the modifying process have taken place successfully. After the modification’s first step (the AA introduction), WCA value was reduced to 65.1 ± 5.3°. The conjugation with peptides resulted in further reduction of WCA values (PU_REDV: 43.9 ± 8.8°, PU_YIGSR: 43.4 ± 7.3°, respectively (statistically significant differed (*p* < 0.05))). 

### 2.6. COOH Group Determination

**Figure 6:**
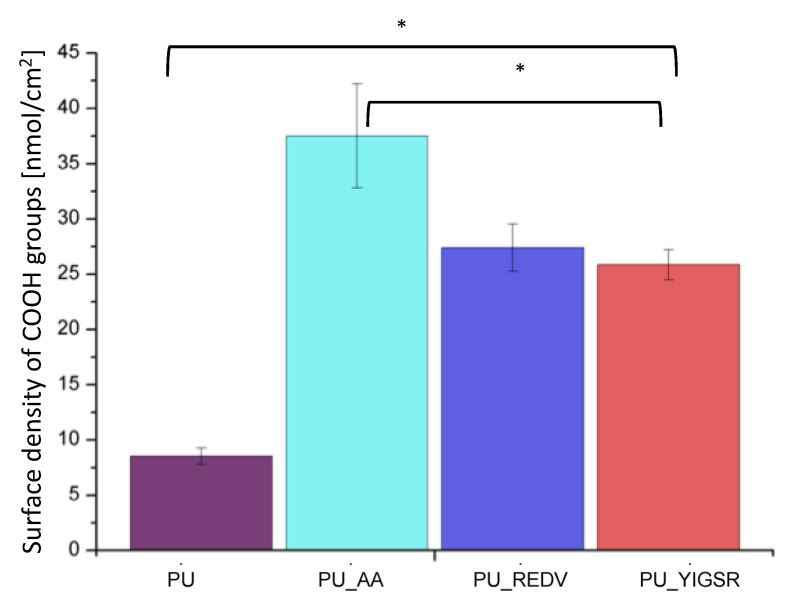
Results of TBO assay (MV ± SD, n = 4), *: *p* < 0.05.

The TBO assay confirms the introduction of carboxyl groups to the surface by the proposed modification method (Figure 6). The unmodified PU gave a slight response in the TBO test (8.5 ± 0.8 nmol/cm^2^). This is due to the fact that the materials’ structure is porous, and the dye has physically deposited on the material. After the first step, the AA introduction, the number of COOH groups increases significantly to 37.5 ± 4.7 nmol/cm^2^ (*p* < 0.05). After the next step, the reaction with the peptides, the number of groups decreased significantly (*p* < 0.05) compared to PU_AA. Which is an expected result as COOH groups were involved in the reaction with the peptide sequences. There is no statistically significant difference between PU_REDV and PU_ YIGSR (24.4 ± 2.1 nmol/cm^2^ and 25.9 ± 1.4 nmol/cm^2^, respectively).

### 2.7. Cytotoxicity

**Figure 7:**
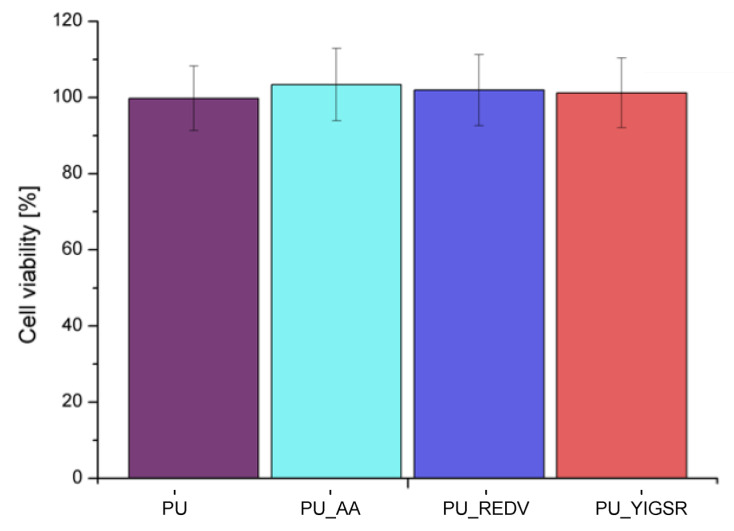
Cell viability (MV ± SD, n = 4).

MTT assay (Figure 7) showed that the viability of cells contacted with all analyzed extracts was close to the control value and significantly exceeded 70%. According to ISO 10993-5 norm, cell viability higher than 70% characterizes non-toxic materials, so it can be concluded that all tested surfaces are biocompatible.

### 2.8. Endothelial and Smooth Muscle Cell Co-Culture

**Figure 8:**
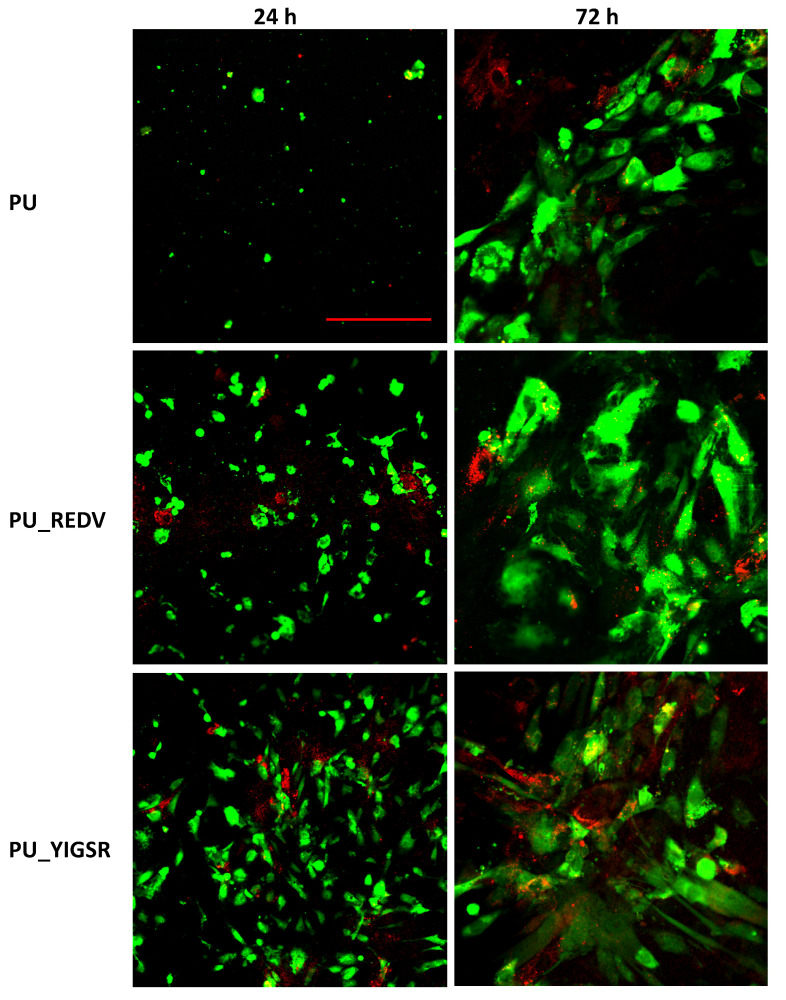
Endothelial cells (green) and smooth muscle cells (red) co-cultured on tested materials, scale bar 100 μm.

EC and SMC co-culture was performed on internal surfaces of the unmodified PU, PU_REDV, and PU_YIGSR for one and three days (Figure 8, Table 2).

After 24 h of culture, single spherical cells were observed on the surface of the pristine PU. Most of them were ECs, only a few SMCs were found. The percentage of the cell-coated area was below 1%. In the case of PU_REDV, a significantly bigger EC-coated area (4 ± 2%) was observed, but the difference was not statistically significant (*p* > 0.05). Cells were evenly distributed on the entire surface. Single SMCs with spherical morphology were observed (<1%). On PU_YIGSR, ECs presented flattened morphology. There very unevenly distributed on the surface; there were gathered in dense clusters, but a cell-free area was also observed. For this surface EC-coated area was the biggest (11 ± 8%), the difference was also statistically significant compared to both PU and PU_REDV. The SMC-coated area was slightly higher compared to PU_REDV, but still very small (<1%).

After 72 h of culture, on unmodified PU, an increase in EC-coated area was observed (8 ± 6%). However, only single SMCs were still visible during observation (SMC-coated area <1%). Both peptide-modified surfaces were densely coated with ECs. Also, an increase in cell-coated area was observed compared to the previous time point. There was no significant difference between both variants of peptide coating, and the ECs-coated area was 10 ± 7% for PU_REDV and 12 ± 3% for PU_YIGSR. A slight increase in the number of surface-adhered SMCs was observed. However, the area was still small (<5%).

### 2.9. Blood-Material Interaction

#### 2.9.1. Platelet Adhesion and Activation under Static and Dynamic Conditions

The hemocompatibility analysis was performed in a flow system. After a 1-h of contact between the prosthesis and the circulating blood, the internal surface of the prostheses was analyzed. No material leakage or soaking was observed. The percentage of area occupied by all platelets (anti-tubulin staining) and activated platelets (anti-CD62P staining) are presented in Table 3. Areas with local clusters of platelets or no platelets on each surface could be observed, resulting in high SD values. For all analyzed variants, the percentage of the area occupied by the platelets was below 5%, with no statistically significant differences (*p* > 0.05). The percentage of the area covered with CD62P-positive platelets was reduced on peptide-modified surfaces compared to the unmodified PU. There were no significant differences between PU_REDV and PU_YIGSR (*p* > 0.05).

**Figure 9:**
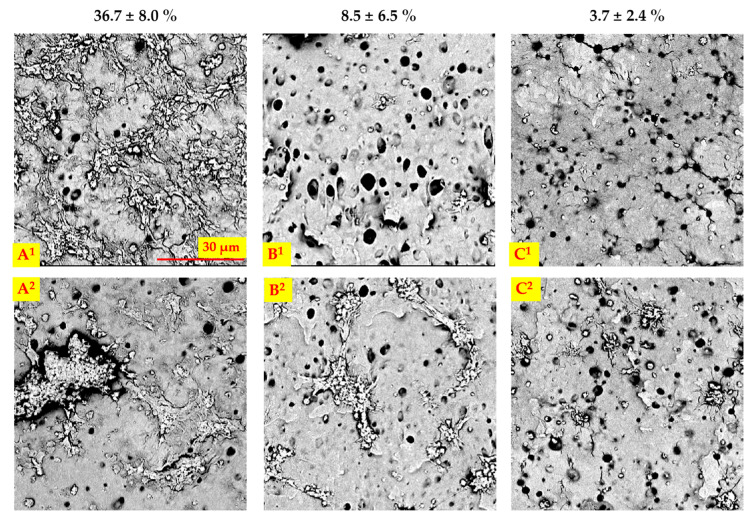
SEM images of analyzed materials’ surface after contact with blood, (**A^1,2^**): unmodified PU, (**B^1,2^**): PU_REDV, (**C^1,2^**): PU_YIGSR, scale bar 30 μm; the percentage of the platelet-coated area is given above each image.

SEM images of materials and the percentage of the surface covered by adhered platelets for each surface variant are presented in Figure 9. Peptide modification significantly reduced the percentage of platelets area (*p* < 0.05), from 36.7 ± 8% for PU to 8.5 ± 6.5% and 3.7 ± 2.4% for PU_REDV and PU_YIGSR, respectively. It proves the positive influence of the suggested peptide sequences on the adhesion of platelets. Platelets of different morphology can be found on all materials tested-round, dendritic platelets and spread dendritic morphology. On unmodified PU, platelet aggregates predominated, and the majority of them had spread dendritic morphology. In contrast, on PU_REDV and PU_YIGSR, platelets appeared singly and had round and dendritic morphology. 

**Figure 10:**
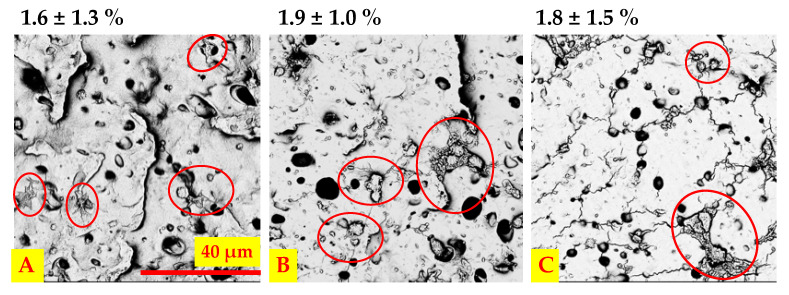
SEM images of analyzed materials’ surface after contact with blood, (**A**) unmodified PU, (**B**) PU_REDV, ad (**C**) PU_YIGSR, scale bar 40 μm; the percentage of the platelet-coated area is given above each image.

After static contact with blood, the percentage of surface area covered with platelets was low and below 5% for all tested materials (no significant difference *p* > 0.05) (Figure 10). The adhered platelets showed varied morphology (i.e., round, dendritic, and spread), with no morphological type predominating over the others.

#### 2.9.2. Whole Blood Clotting Time

**Figure 11:**
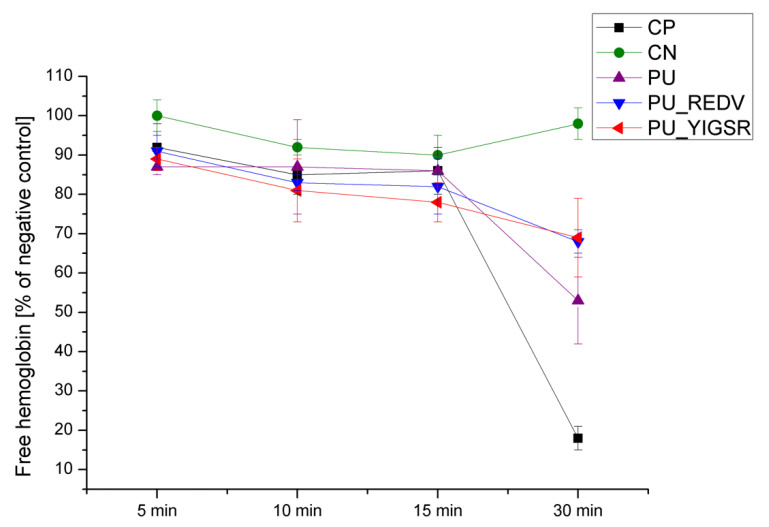
Results of whole blood clotting time assay.

Figure 11 presents the change in the amount of hemoglobin released (absorbance measured at 540 nm) from blood samples contacted with materials for 5, 10, 15, and 30 min. The higher content of free hemoglobin corresponds to a lower amount of clot produced (which means higher hemocompatibility of the material contacted with blood sample).

The negative control (blood without CaCl_2_) did not clot at any time point tested and presented free hemoglobin values ≥90%. In contrast, in the positive control (blood with the addition of CaCl_2_), a clot was formed after 30 min, and the level of free hemoglobin dropped to 18 ± 3%. In the case of analyzed materials, there was no significant difference in the level of free hemoglobin in the first three time points (5 min, 10 min, and 15 min). After 30 min of contact, there were differences in the amount of free hemoglobin present in blood samples contacted with the materials. Blood contacted with pristine PU had free hemoglobin of 53 ± 11%. Blood contacted with the peptide-modified materials had a significantly (*p* < 0.05) higher free hemoglobin content: 68 ± 3 and 69 ± 10%, respectively, for PU_REDV and PU_YIGSR. There was no statistically significant (*p* > 0.05) difference between values for PU_REDV and PU_YIGSR at that time point.

## 3. Discussion

The following studies are a continuation of the works presented in our previous paper [31]. In that paper, parameters for the production of cylindrical structures by the phase inversion were selected. The following work presents the material modified with a peptide coating and analyzes the influence of the applied modification on the properties of the prostheses, with the main focus on its bio- and hemocompatibility.

Surface modification is an integral part of work on vascular prostheses. Bioactive molecules’ introduction simultaneously changes surface hydrophilicity, which is reported to enhance cell adhesion [38]. Moreover, the use of appropriate peptides promotes the adhesion and growth of endothelial cells, naturally occurring in blood vessels. Therefore, in the research described here, it was decided to use the REDV and YIGSR peptide sequences. These are one of the most often studied EC-selective sequences [39]. A crucial aspect of the modification is also its effect on the blood-material interaction. Immediately after implantation of the material, the nonspecific adsorption of various proteins from serum and water occurs [40]. As the scaffold will be in constant and direct contact with blood, it cannot increase clotting or platelet adhesion. That is also the reason why changing the wettability of the surface is such an important issue [41].

Each step of the surface modification reaction was monitored by the FTIR and XPS. The analysis is consistent with the results obtained on materials obtained via different methods (solvent casting, solution blow spinning) with varying reaction parameters [31,42], confirming that the reaction occurred. This proves that it is possible to introduce acrylic acid and peptide sequences on material produced by the phase inversion technique. The reaction is also confirmed by a change in wettability. The grafting of carboxyl groups alone reduced the CA, and the introduction of peptide sequences further increased the surface hydrophilicity. This is a fully expected result [41,43]. Colorimetric analysis with TBO correspondingly confirmed the occurrence of the reaction. After reacting with the peptide sequences, the number of COOH groups on the surface decreased as they engaged in the reaction. The response of materials after modification with the peptide sequences is an expected result, as all peptides have carboxyl groups in their structures.

An increase in pore diameter of the internal surface was observed after the reaction with AA. With a different surface activation method, Seman et al. proved that AA grafting could increase the pore diameter [44]. Similarly, Shi et al. showed an increase in pore diameter after a reaction with AA [45]. The increase in pore diameter is most likely due to surface activation with cerium ions, a short reaction time, and low AA concentration. In the event of a longer time and higher AA concentration, the diameter of surface pores would likely decrease. However, such surface pores in small-diameter vascular prostheses, according to Camper et al. [46], should have higher patency rates and the development of thinner neointima. Moreover, the range of pore sizes shown by the obtained materials should favor the growth of EC [47]. The surface modification increased the porosity of the material without changing the surface morphology. This is a favorable phenomenon that should allow for better tissue overgrowth [48]. For the PU_AA, the porosity was higher than for the peptide materials (statistically insignificant difference). The reduction of porosity is related to the modification steps. 

The YM increase is in line with previous research on fibrous material [42]. The increase in YM was observed after modification with the REDV peptide, similar to the studies described here. Compared to those studies, the tensile strength did not increase that much. On the other hand, elongation at the break here decreased. Those results prove how the mechanical properties depend on the very structure of the material. The modification slightly influenced the mechanical properties; however, the values of the analyzed parameters are within the ranges appropriate for vascular prostheses [49].

The use of the described modification did not affect the viability of L929 cells that were contacted with the material extracts. An EC and SMC co-culture was performed on unmodified and peptide-modified materials, where both cells’ types were in close contact. As a result, it was possible to evaluate the competitive attachment and growth between endothelial and smooth muscle cells. Cells culture was carried out for three days due to the dyes used for the observation (imaging of live cells). On the first day, on the unmodified material, the ECs were not flattened, while on the peptide-modified materials, there were more of them, with flattened morphology. This proves the positive effect of the used peptides. On PU_REDV, ECs were found on the entire materials surface, while on PU_YIGSR, they were densely clustered, and the rest of the materials remained cell-free. Despite seeding both types of cells in the same number, the number of ECs cells was significantly higher than the number of SMCs. On the second day, the cells increased their number. Spherical and flattened cells were visible on the PU. On PU_REDV and PU_YIGSR, EC significantly increased their occupied area, showing that the introduced peptide positively affects their adhesion and growth promotion. Such an effect on EC was also observed on the third day on both peptide-modified materials. This is fully confirmed in the literature [50,51]. There is also an increase in the area occupied by the SMC, but it is a slower process than in the case of the EC. On the first day, SMC appeared singly on all materials. In the following days of cell culture, they began to adopt their typical elongated morphology. However, on each analyzed culture day and each type of material, the number of SMCs was significantly lower compared to the number of ECs.

In the case of blood-material tests, it was decided that we would perform an hour-long contact of the material with blood based on literature reports [52,53]. Tubulin is important for platelet mobilization, reshaping, and formation of pseudopod [54]. P-selectin (CD62P) is a marker of platelet activation. After activation, P-selectin moves to the platelet surface, and the P-selectin-specific antibody binds exclusively to the activated platelets [55]. Blood analyzes showed that both platelet adhesion and activation were low. Platelet adhesion and activation indicate the prevalent mechanism by which biomaterial thrombogenicity occurs [8]. Zhang and Shen demonstrated that increasing hydrophilicity favors platelet adhesion [56]. In addition, the introduction of peptides to the surface may increase platelet adhesion. Our research showed that both REDV and YIGSR peptides reduced the percentage of the area covered with adhered or activated (CD62P-positive) platelets. Thus, promoting effect of the introduced peptide on platelet adhesion was found, as confirmed by other authors [9,12]. It should be emphasized that in the studies, medical-grade polyurethane with low thrombogenicity was used, hence why there was such a low percentage of platelets on all tested materials. Morphology of the surface-adhered platelets was varied. However, platelet spread in large aggregates prevailed on unmodified PU, forms typical for activated platelets [55]. On the peptide-modified surfaces, round and single dendritic platelets dominated, characteristic for inactive plates. On the other hand, there are differences in the percentage of the area occupied by platelets and their morphology on the material’s surface after dynamic and static flow. This is due to the nature of the contact. When the shear stress is removed, the blood behaves like an elastic solid. Therefore, in the case of static, the adhesion of platelets was most affected by the surface’s wettability. Hence the similar blood platelet counts and their morphologies on all materials. As already mentioned, medical-grade polyurethane was used; therefore, no significant differences between analyzed samples in static contact could be expected. During dynamic contact, additional forces (shear stress) due to the flow acted on the blood that activated platelets. The flow system was more like the natural environment inside the blood vessels that favors the activation of platelets. This result proves the influence of peptide sequences on the behavior of platelets.

In order to assess the activation of the coagulation mechanism, the whole blood clotting time test was used. The test evaluates the level of free hemoglobin released from red blood cells that were not trapped in the clot formed during blood-material contact. The difference in the free hemoglobin level appeared after 30 min of incubation. Pristine PU had a significantly lower free hemoglobin level compared to the peptide-modified materials. This result confirmed the positive influence of the introduced coating on surface hemocompatibility, which is in line with the literature data [25].

In summary, the method of obtaining and modifying cylindrical polyurethane structures obtained via phase inversion was presented. These materials are designed for use as vascular prostheses. It has been shown that the introduced peptide coating increases the adhesion of EC cells while maintaining low adhesion of SMCs. The introduced peptides did not increase platelet adhesion and activation. At the same time, a positive effect on the amount of free hemoglobin present in the blood contacted with the analyzed surface was demonstrated.

## 4. Materials and Methods

### 4.1. Materials

Polyurethane, ChronoFlex C45D, was bought in the form of pellets from AdvanSource Biomaterial, Wilmington, MA, USA. N,N-dimethylacetamide (DMAC), acrylic acid (AA, 99%), sodium dodecyl sulphate (SDS, 98%), 1-ethyl-3-(3-dimethylaminopropyl) carbodiimide (EDC, purity ≥ 98.0%), MES buffer (2-(N- morpholino)ethanesulfonic acid, ≥99.5%), phosphate-buffered saline (PBS, tablets), toluidine blue O (TBO), sodium hydroxide (NaOH), acetic acid, hexamethyldisilazane (HMDS), ethanol(EtOH, 99%) were purchased from Sigma Aldrich, Poznań, Poland. Nitric acid (HNO_3_, 65%) was purchased from Carlo Erba, ammonium cerium (IV) sulphate dihydrate ((NH4)4Ce(SO4)4.2H2O, p.a. grade) from Riedel-de Haen). Peptides with GSGREDVGSG (REDV) and GSGYIGSRGSG (YIGSR) sequences (purity ≥ 98%) were purchased from Novazym, Poznań, Poland (REDV, 99.52%). N-hydroxysulfosuccinimide (sulfo-NHS, purity ≥ 98.0%) was purchased from ThermoFisher, Waltham, MA, USA. 

### 4.2. Materials Preparation

#### 4.2.1. Preparation of Polyurethane Scaffolds

The cylindrical scaffolds were obtained using the phase inversion method with ethanol as the non-solvent. This process was described in detail in our previous work [30]. Briefly, PU granules were first purified with 70% EtOH solution and dried at 40 °C. A 20% *w/v* PU/DMAC solution was prepared and mixed on a shaker until the polymer was completely dissolved. After that, the metal collector (⌀ 6 mm) was immersed in a polymer solution. Subsequently, the collector coated with polyurethane solution was immersed in the non-solvent (0:100 water/EtOH) for 24 h; the process was carried out at 50 °C. After this time, the scaffolds were removed from the collector and dried at room temperature (RT) with increased humidity.

#### 4.2.2. Surface Modification

The obtained scaffolds were modified with acrylic acid, followed by conjugation with peptides. The samples subjected to the modification had the shape of cylinders with an internal diameter of 5 mm and a length of 40 mm. Materials were placed individually in test tubes at each step of the reaction. Samples were fully immersed in a modifying solution with a volume of 5 mL (to have a constant ratio of surface area to the solution volume) and placed on a roller mixer for the reaction time (to provide a constant flow of modification solutions through the inner part of the materials). The modification reaction was performed in two steps. In the first step, samples were incubated in a 2.3% (*v/v*) solution of nitric acid in distilled water solution, to which was added (NH_4_)_4_Ce(SO_4_)_4_ (final concentration = 0.01% *w/v*) and AA (final concentration = 1% *v/v*). The reaction was carried out for 30 min at RT. Then, the materials were rinsed with SDS solution (0.1% *w/v*) and water for 20 min. Materials after this modifications step were marked as PU_AA. In the second modification step, samples were incubated in an MES buffer (0.05 M, pH = 6.0) for 1 h, RT. After that time, materials were transferred to the solution of sulfo-NHS (5 mM) and EDC (2 mM) in MES buffer, pH = 6.0, and incubated for 15 min. Materials were rinsed with MES buffer and then incubated in the peptide solution (GSG**REDV**GSG or GSG**YIGSR**GSG, 20 μg/mL in PBS, pH = 8) for 1 h, RT. Afterward, samples were rinsed with PBS, pH = 8, followed by rinsing with water. Materials after reaction with REDV or YIGSR peptides were marked as PU_REDV, PU_YIGSR, respectively.

### 4.3. Physicochemical Properties

#### 4.3.1. Surface Morphology

Samples’ morphology was analyzed using a scanning electron microscope (SEM, Phenom G1, Phenom World, Eindhoven, The Netherlands). Rectangular fragments were cut from each cylindrical structure (n = 4). The internal surfaces were examined, and images from randomly selected spots were captured. Surface pore sizes (n = 100) were measured with ImageJ based on SEM images [57].

#### 4.3.2. Chemical Characterization 

FTIR spectra were recorded with Nicolet 6700 Smart Orbit Diamond ATR (Thermo Scientific, Waltham, MA, USA) and analyzed with the use of the Omnic 8.3 software. The materials were cut open and tested to analyze the inner surface. Spectra were recorded in at least three randomly selected spots, and each material was prepared in triplicate. 

X-ray photoelectron spectroscopy (XPS) analysis was conducted using Scienta R4000. Spectra fitting and determination of atomic composition were obtained with the CasaXPS software.

#### 4.3.3. Wettability

The wettability of the materials was analyzed after each step of modification using a DSA100 goniometer (Krüss GmbH, Hamburg, Germany). Flat square fragments of cylindrical structures (n = 4) were cut off and glued to a glass slide. A droplet of distilled water (5 μL) was placed on each material, and water contact angle (CA) was measured automatically with DSA100 goniometer’s software. 

#### 4.3.4. Carboxyl Groups Determination

In order to calculate the surface density of COOH groups, a toluidine blue (TBO) assay was performed using a protocol described elsewhere [58]. Square fragments (sample size: 1 cm × 1 cm, n = 4) were placed separately in a 500 μL of TBO solution (0.5 mM, pH 11) for 3 h at RT. After the incubation, samples were gently flushed with water, paper-dried, and placed in 500 μL of 50% acetic acid solution (50% *v/v*, pH 1.5) for 15 min, RT. After the incubation time, the absorbance of the solutions was measured at 633 nm using a plate reader. The surface density of COOH groups was calculated based on the standard curve. 

### 4.4. Porosity

The porosity of the materials was determined by the gravimetric method [59,60]. Prosthesis (cylindrical) was cut into 20 mm in length and weighed (n = 4). The porosity of materials was calculated based on its apparent density (ρ_app_) and known density of the polymer (ρ_p_ = 1.2 g/cm^3^ [61]), in accordance with the formula:

porosity [%] = (1 − ρ_app_/ρ_p_) × 100%

where values of ρ_app_ were measured from the weight and dimensions of the materials (n = 4). 

### 4.5. Mechanical Testing

Cylindrical samples (5 mm inner diameter, 60 mm length; n = 4) were subjected to uniaxial tensile testing according to protocols based on ASTM standards (882-02 and D 638-02a). The study was performed using the Instron 3345 model with 5 mm/min head speed at RT and ambient humidity.

### 4.6. Cytotoxicity 

The cytotoxicity study, MTT assay (Thiazolyl Tetrazolium Blue Bromide, Sigma-Aldrich, Poznań, Poland), was performed according to ISO 10993-5 [62]. L929 cells were grown in Dulbecco Modified Eagle Medium (DMEM, ThermoFisher, Waltham, MA, USA) supplemented with bovine serum (10% *v*/*v*, ThermoFisher, Waltham, MA, USA) mixture of penicillin-streptomycin antibiotics (1% *v/v*, ThermoFisher, Waltham, MA, USA) in an incubator (37 °C, 5% CO_2_).

Materials (cylindrical fragment) were sterilized with a solution of penicillin-streptomycin (100 U/mL, GibcoTM, ThermoFisher, Waltham, MA, USA) and 0.1% (*v/v*) amphotericin B (GibcoTM, ThermoFisher, Waltham, MA, USA) in sterile PBS at 4 °C for 1 h. Next, samples were washed five times with sterile PBS on a plate shaker for 5 min. Then, materials were incubated in DMEM for 24 h to obtain the material’s extracts. Cells were seeded in a 96-well plate at a 1 × 10^5^/mL density and cultured for 24 h. Next, material extracts were added to the cells and incubated for 24 h. Cells incubated with DMEM and cells incubated with 0.1% Triton X in DMEM were used as a negative and positive control, respectively. On the next day, a solution of MTT (1 mg/mL DMEM) was added to the wells. The plates were incubated for 4 h. Then, the solution was removed, and formazan crystals were dissolved in isopropanol. Absorbance was measured at λ 570 nm.

Cell viability was calculated from following formula:

cell viability [%] = A_S_/A_C_ × 100%

where: A_S_ is the sample mean absorbance value, A_C_ is the negative control mean absorbance value. 

### 4.7. Endothelial and Smooth Muscle Cell Co-Culture

Human Umbilical Vein Endothelial Cells (EC, Lonza, Basel, Switzerland) were cultured in Endothelial Cell Growth Medium-2 (EGM-2, Lonza, Basel, Switzerland). Smooth Muscle cells (SMC, Lonza, Basel, Switzerland) were cultured in Smooth Muscle Cell Growth Medium 2 (PromoCell, Heidelberg, Germany); both media were supplemented according to the manufacturer’s instructions. All cells were grown in an incubator at 37 °C, 5% CO_2_.

Materials (flat squares) were sterilized in a mixture of antibiotics as described in Section 4.6.

Before cell seeding, EC and SMC cells were stained in culture flasks with CellTracker™ Green (EC) and CellTracker™ Red (SMC) dyes (Invitrogen, Waltham, MA, USA) according to the manufacturer’s instructions. Briefly, the medium was removed from the culture, 1.5 mL of dye solution was added to the flask and incubated for 30 min in an incubator. After removing the dye, cells were harvested using trypsin. Then cells were mixed in a 1:1 ratio and seeded on sterilized materials (total cell density 1.0 × 10^5^/mL). After culture, the microscope slides were prepared for observation under the confocal microscope. 

### 4.8. Interaction with Blood

Whole blood was collected in 1.8 mL test tubes containing citrate (BD Vacutainer, Franklin Lakes, NJ, USA) from a healthy woman. The blood was brought from the Center for Blood Donation. Blood was immediately used in the experiments.

#### 4.8.1. Blood-Material Interaction

For dynamic testing, the samples were cylinders with an internal diameter of 5 mm and a length of 40 mm. Each sample was contacted with 5 mL of whole blood circulating in a flow system with a 20 mL/min flow for 1 h as recommended by the other authors [52]. After this time, the materials were rinsed with 0.9% NaCl and further analyzed. The materials were cut open and subjected to further analysis.

For tubulin staining materials were fixed in the CytoFix/CytoPerm Kit (BD Biosciences, Franklin Lakes, NJ, USA) for 20 min at 4 °C. The samples were then washed with Wash Buffer and incubated in 0.1% Bovine Serum Albumin (BSA)/PBS for 60 min. Samples were washed in PBS, 3 × 5 min. After that, the materials were incubated with the alpha Tubulin Monoclonal Antibody eFluor 615 (eBioscience ™ (Waltham, MA, USA), 1: 200 in PBS) in the dark at RT for 1h. After this time, the materials were washed in PBS, 3 × 5 min. The materials were glued to the cover glass with ProLong Gold antifade mountant (Thermo Fisher, Waltham, MA, USA) and left for analysis. For the CD62P staining, materials were incubated with the primary antibody, Recombinant Anti-CD62P (1: 500 in 1% BSA/PBS), for 2 h, RT. The samples were then washed very thoroughly with PBS, 3 × 5 min. Afterward, the materials were incubated with Goat Anti-Rabbit IgG H&L secondary antibody (Alexa Fluor^®^ 488, Thermo Fisher, Waltham, MA, USA) (1: 1000 in 1% BSA/PBS) for 1h, in the dark, RT. The materials were then rinsed with PBS, 3 × 5 min, glued to the cover glass with the ProLong Gold antifade mountant, and left for analysis. For SEM analysis, materials were fixed in 4% PFA, rinsed with PBS, and dehydrated in an increasing EtOH series (50%, 60%, 70%, 80%, 90%, 100%, 10 min each). The materials were then incubated with a 1:2 EtOH/ hexamethyldisilazane (HMDS) mixture and left for 20 min. After this time, the materials were transferred to a 2:1 EtOH/HMDS mixture. After 20 min, the materials were flooded with HDMS solution and left for the next 20 min. Finally, samples were left to dry overnight, coated with a 15 nm layer of gold/palladium alloy (80/20 at. %), and subjected to SEM observation (SEM, Phenom G1, PhenomWorld, Eindhoven, The Netherlands). The percentage of the platelet-coated area was calculated using Fiji software.

Samples were also contacted with blood under static conditions. Each material (flat square, 0.5 cm × 0.5 cm size) was incubated with whole blood (500 μL) at 37 °C for 1 h, followed by a procedure for SEM analysis described above.

#### 4.8.2. Whole Blood Clotting Time

The antithrombogenicity of obtained materials was assessed using a whole blood kinetic clotting time method described elsewhere [63,64]. The cylindrical materials were cut open, and samples were prepared in the form of square pieces with dimensions of 5 × 10 mm. Three replicates were used for each time point. 1 mL of 0.1 M CaCl_2_ solution was added to freshly drawn blood (10 mL) to activate the blood coagulation mechanism. A well of a polystyrene plate with activated or inactivated (without CaCl_2_) blood was used as a positive (CP) or negative control (CN), respectively. The test was carried out in static conditions by placing 50 μL of activated blood on each material. Incubation (5, 15, 30 min) was carried out at RT. After each time point, 1.5 mL of water was added to the material and incubated for the next 5 min. Next, 200 μL of the obtained solution was transferred to a 96-well plate, and absorbance was measured at 540 nm. 

The percentage of free hemoglobin was calculated from the following formula:

Free hemoglobin [%] = A_S_/A_CN5_ × 100%

where: A_S_ is the sample mean absorbance value, A_CN5_ is the negative control mean absorbance value after 5 min of blood-material contact.

### 4.9. Statistical Analysis

The results were expressed as mean values ± SD. Statistical significance of differences was investigated with a single-factor analysis of variance (ANOVA) for *p* < 0.05 with post-hoc Tukey’s test (OriginPRO 8.0, OriginLab Corporation, Northampton, MA, USA).

## 5. Conclusions

Cylindrical vascular scaffolds were prepared from polyurethane solution using a simple phase inversion technique. Next, acrylic acid conjugated with REDV/YIGSR peptide were successfully introduced to the prosthesis’ surface. Peptide-modified internal surfaces have a pore size in the range of 1–11 μm with an average pore size of approximately 5 μm. The obtained prostheses had a porosity of about 60%, Young’s Modulus in the range of 9–11 MPa, and a water contact angle around 40°. MTT assay demonstrated that all analyzed materials were not cytotoxic. Peptide modification contributed to enhanced adhesion and proliferation of ECs on the internal surface. At the same time, the peptide-modified surfaces promoted adhesion of ECs over SMCs, as confirmed by the co-culture of both cell types. Analysis of blood-material interactions confirmed the high level of prostheses hemocompatibility. It has been shown that the applied peptide coating significantly reduced the amount of clot formed during blood-material contact. The study of platelet adhesion under flow conditions showed that the surface area occupied by the platelets on the REDV-coated prosthesis was 4 times smaller, and on the YIGSR-coated prosthesis, 10 times smaller compared to the prosthesis without peptide coating. Considering the above results, it can be concluded that the peptide coating increased the hemocompatibility of the surface by increasing ECs adhesion and, at the same time, decreasing platelet adhesion. Comparing both types of peptide coatings, more promising results were obtained for the surfaces coated with the YISGR than the REDV-coated prostheses.

## Figures and Tables

**Table 1:** Psychical and mechanical properties of obtained materials (MV ± SD).

Material	PU	PU_AA	PU_REDV	PU_YIGSR
wall thickness [μm]				
	230.8 ± 41.5	228.6 ± 35.4	227.1 ± 34.0	229.9 ± 33.2
average surface pore diameter [μm]		*	*	*
	3.0 ± 1.0	3.5 ± 1.4	4.4 ± 1.5	4.6 ± 1.9
min surface pore diameter [μm]				
	1.0	1.4	1.4	1.6
max surface pore diameter [μm]				
	5.8	9.0	9.5	10.1
porosity [%]		*	*	*
	56 ± 2	65 ± 5	62 ± 6	63 ± 3
Young’s modulus [MPa]		*	*	*
	3.6 ± 1.5	9.1 ± 1.7	9.8 ± 1.8	10.6 ± 2.4
tensile strength [MPa]		*	*	*
	11.2 ± 1.2	3.8 ± 1.1	5.7 ± 0.7	6.7 ± 1.1
elongation at break [mm/mm]		*	*	*
	4.7 ± 0.4	1.4 ± 0.5	1.9 ± 0.3	2.1 ± 0.4

*: *p* < 0.05 vs. PU.

**Table 2:** Percent of the area occupied by EC and SMC.

		PU	PU_REDV	PU_YIGSR
Area coated with ECs [%]	1 day	<1	4 ± 2	11 ± 8 *^,#^
	3 day	8 ± 6	10 ± 7	12 ± 3
Area coated with SMCs [%]	1 day	<1	<1	<1
	3 day	<1	2 ± 2	3 ± 3

* *p* < 0.05 vs. PU, # *p* < 0.05 vs. PU_REDV.

**Table 3:** Analysis of blood-material interaction in flow system.

	PU	PU_REDV	PU_YGSR
% of area covered with platelets (anti-tubulin staining)	3.2 ± 2.7	4.3 ± 8.8	3.8 ± 5.2
% of area covered with activated platelets (CD62P staining)	0.4 ± 0.5	0.1 ± 0.1	0.1 ± 0.1

## Data Availability

Not applicable.
